# New ferrocene modified retinoic acid with enhanced efficacy against melanoma cells *via* GSH depletion†

**DOI:** 10.1039/c8ra04078h

**Published:** 2018-08-03

**Authors:** Yibo Wang, Bin Sun, Bin Han, Min Hu

**Affiliations:** Department of Orthodontics, School and Hospital of Stomatology, Jilin University Changchun 130041 P. R. China MinHu68@sohu.com; Jilin Provincial Key Laboratory of Tooth Development and Bone Remodeling, Jilin University Changchun 130041 P. R. China; Department of Oral and Maxilloficial Surgery, School and Hospital of Stomatology, Jilin University Changchun 130041 P. R. China

## Abstract

Malignant melanoma is a highly lethal disease, and advanced stages of melanoma have proven to be resistant to many chemotherapeutic drugs. Cancer stem cells (CSCs) and high levels of intracellular glutathione (GSH) have been proven to play important roles in drug resistance. Retinoic acid (RA) is a promising anticancer agent, which can inhibit proliferation and induce differentiation of CSCs, but its clinical use has been limited by its water insolubility and weak cancer cell killing effect when used alone. Herein, by combining RA and ferrocene, a new type of derivative of retinoic acid (FCRA) was synthesized and then oxidized by FeCl_3_. The oxidized FCRA (FCRA^+^) was exploited as a novel anticancer agent. Compared with RA, FCRA^+^ not only has improved water solubility and stronger anti-cancer effect to melanoma cells through depleting intracellular GSH of the cancer cells, but also can inhibit proliferation and induce differentiation of melanoma CSCs, such as free RA. Therefore, FCRA^+^ has better application prospects than RA and may replace RA for clinical applications.

## Abstract 2

Malignant melanoma is a highly lethal disease, and advanced stages of melanoma have proven to be resistant to many chemotherapeutic drugs.

## Graphical abstract

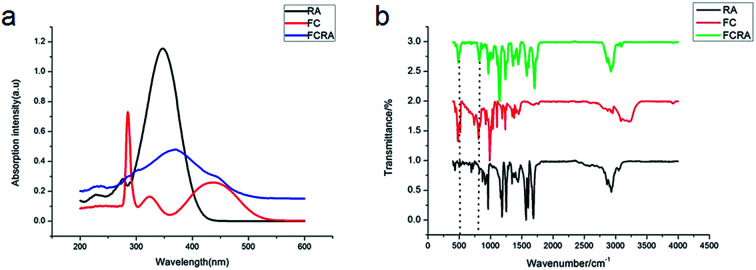

## 1. Introduction

According to the World Health Organization, malignant melanoma remains among the most notoriously aggressive human cancers, and its incidence has increased drastically over the last few decades.^1,2^ Despite various types of treatments, advanced stages of melanoma have proven to be resistant to many therapies. Multiple mechanisms contribute to the development of drug resistance in melanoma.^3,4^ CSCs in advanced stage of melanoma are one of the main reasons for drug resistance. CSCs are a small amount of undifferentiated or poorly differentiated cancer cells. Higher expression of intracellular and extracellular drug transport systems and stronger ability of proliferation, anti-apoptosis and damage repair of DNA of CSCs make them more tolerant to chemotherapeutic drugs than other differentiated cancer cells, namely, non-CSCs.^5,6^

CSCs targeted drugs are effective in inhibiting cancer drug resistance,^7^ but a significant limitation of CSCs targeted drug design is that complex synthetic schemes are often required, resulting in low loading and higher barriers to clinical translation. Retinoic acid (RA) is a small lipophilic molecule derived from vitamin A. Retinoic acid is a promising anticancer agent, and the anticancer activity was first discovered in the treatment of acute promyelocytic leukemia (APL), which has been confirmed in various tumor types at present.^8–10^ RA can induce undifferentiated or poorly differentiated CSCs into differentiated cancer cells, reduce the proliferation and migration ability of CSCs, and increase the sensitivity of CSCs to chemotherapeutic drugs.^11,12^ However, retinoic acid is insoluble in water and the capacity of inducing cancer cell apoptosis is poor when used alone, which limits its clinical use.^13^

Moreover, it has been fully proved that increased content of intracellular GSH in cancer cells, particularly CSCs, is another common reason for drug resistance,^14,15^ making cancer cells have stronger ability of detoxification and eliminating oxygen free radicals caused by chemotherapeutic drugs. However, the consumption of intracellular GSH can inhibit the drug resistance of cancer cells and improve the therapeutic effect of anticancer drugs.^16^

Ferrocene is a neutral, chemically stable, nontoxic molecule. Because of its excellent redox properties, it has attracted special attention in drug research.^17^ Many ferrocenyl compounds exhibit interesting cytotoxicity and anti-tumor activities.^18–20^ Ferrocene can be oxidized to ferrocene cation, which is sensitive to GSH, and can be reduced to ferrocene by GSH.^21^ With the consumption of GSH in cancer cells, the detoxification ability and antioxidant capacity of cancer cells were decreased, and the resistance to therapeutic drugs was reduced. Herein, we report the synthesis and biological evaluation of new ferrocene modified retinoic acid with covalent binding of retinoic acid to ferrocenylmethanol (FC), which was then oxidized by FeCl_3_. The developed derivative FCRA^+^ not only has improved water solubility and stronger anti-cancer effect than free RA to melanoma cells, but it also retained the ability of inducing melanoma CSCs into differentiated melanoma cancer cells such as free RA.

## 2. Experimental section

### 2.1 Materials

All *trans* retinoic acid (ATRA), ferrocenylmethanol (FC), Hoechst 33342, verapamil hydrochloride and Paclitaxel were obtained from Sigma-Aldrich reagent company. Diisopropyl azodicarboxylate, diisopropyl azodiformate (DIAD), and triphenylphosphine (PPh_3_) were obtained from Aladdin reagent company. Tetrahydrofuran (THF) was purchased from Beijing Chemical Works.

### 2.2 Synthesis of ferrocenyl retinoic acid (FCRA)^22^

ATRA (3 mmol), ferrocenyl-methanol (3 mmol), and PPh_3_ (4.5 mmol) were dissolved in THF (20 mL). Then, the stirred solution was added to DIAD (4.5 mmol) under N_2_ atmosphere at 0 °C. The reaction mixture was allowed to warm to room temperature and stirred for additional 2 h. The reaction was monitored by TLC. The yellow reaction mixture was concentrated on a rotary evaporator (30 °C) to obtain a viscous oil. The residue was purified by column chromatography (ethyl acetate/petroleum ether, v/v = 2 : 8) and FCRA was obtained in the yield of 92%.

### 2.3 Preparation of oxidized FCRA (FCRA^+^)

For preparation of oxidized FCRA, 0.1 mmol FeCl_3_ was dissolved in 1 mL CH_3_CN and 0.01 mmol of FCRA was dissolved in 9 mL CH_2_Cl_2_. Then, they were mixed and FCRA was oxidized by FeCl_3_. The reaction ratio ensures that all the ferrocenyl groups of FCRA are oxidized. Following this, the solvent was removed under vacuum and FCRA^+^ was purified by dialysis (MW = 3500).

### 2.4 Characterization

UV-vis absorption spectra were obtained using a Shimadzu 3100 UV-vis spectrophotometer. IR spectra were recorded on a Nicolet AVATAR 360 FT-IR spectrophotometer. ^1^H-NMR was obtained using AVANCEIII 500.

### 2.5 Cell cultures

Human melanoma cell line A375 was acquired from ATCC. Cells were grown in Dulbecco's modified Eagle's medium (DMEM) supplemented with 10% newborn calf serum (GIBCO), 4 mM glutamine, 100 U mL^−1^ penicillin and 100 mg mL^−1^ streptomycin.

### 2.6 Cell viability assay

A375 cells (5 × 10^3^) were seeded in 96-well plates cultured for 24 h in an incubator (37 °C, 5% CO_2_) and for another 24 h after the culture medium was replaced with 150 μL of DMEM medium containing the RA and FCRA^+^ at different doses (0, 5, 10, 20, 40, 80 μM). Then, 20 μL of 5 mg mL^−1^ working concentration of MTT solution was added to every cell well. The cells were further incubated for 4 h. Finally, 150 μL of DMSO was replaced for the medium of each well to dissolve formazan crystals and then, the absorbance was obtained at a test wavelength of 490 nm. The cell viability was calculated by the following formula: percentage cell viability = (absorbance of the experiment samples/absorbance of the control) × 100%.

### 2.7 Annexin V-FITC/PI staining

Annexin V-FITC and PI double staining were employed to determine apoptosis. In brief, A375 cells (2 × 10^5^) were seeded in 6-well plates, treated by the same dose (20 μM) of RA and FCRA^+^ for 24 h, and then stained with KeyGen Annexin V-FITC apoptosis detection kit according to the manufacturer's protocol. Then, apoptosis was assessed using a FACSCalibur Flow cytometer. Fluorescence was measured with the excitation wavelength of 480 nm and the emission wavelength of 578 nm through FL-1 filter and FL-2 filter.

### 2.8 Determination of intracellular GSH and ROS levels

A375 cells (2 × 10^5^) were seeded in 6-well plates, treated by the same doses (0, 5, 10, 20, 40, 80 μM) of RA and FCRA^+^ for 24 h. Intracellular GSH and ROS levels were measured by staining with NDA and DCFH-DA.^23–25^ The cells were stained with 200 μM NDA and 10 μM DCFH-DA separately at 37 °C for 30 min. Then, the cells were washed with PBS, and the level of GSH and ROS were determined by measuring the fluorescence intensity by flow cytometer through the FL-1 filter with an excitation wavelength of 480 nm.

### 2.9 Sorting of SP cells

A375 cells were harvested and pre-warmed at 37 °C for 10 min. Then, the cells were labeled with Hoechst 33342 at a concentration of 5 μg mL^−1^ with or without adding 100 μM verapamil hydrochloride in the same medium at 37 °C for 90 min; verapamil served as an inhibitor of verapamil-sensitive ABC transporters.^26^ Furthermore, the analysis and sorting were performed on a FACSDiva (Becton Dickinson, San Jose, CA, USA) by using dual wavelength analysis (blue, 420–470 nm; red, 660–680 nm).

### 2.10 Effect on differentiation of SP cells

The SP cells from flow sorting were seeded in 6-well plates (2 × 10^5^), using DMEM medium containing 2% newborn calf serum for 24 h. Then, the culture medium was replaced with serum-free DMEM medium containing the same dose of RA and FCRA^+^ (20 μM) for another 6 h. The cells were continued to be cultured for 48 hours using DMEM medium containing 2% newborn calf serum and then, the cells were harvested. The RNA was extracted according to the RNA extraction kit, and the RNA concentration was detected by NanoDrop 1000 spectrophotometer. Then, RNA was transcribed to cDNA according to the PrimerScript RT Reagent Kit instructions, and real-time PCR was used to detect the expression changes of Oct-4 and Sox2.

### 2.11 Effect on sensitivity of SP cells to paclitaxel

The SP cells and non-SP cells (5 × 10^3^) were sorted and seeded in 96-well plates and cultured for 24 h in an incubator (37 °C, 5% CO_2_). The SP cells were cultured using DMEM medium containing 2% newborn calf serum for 24 h, and the non-SP cells were cultured using DMEM medium containing 10% newborn calf serum. To detect the sensitivity of the SP cells and non SP cells to paclitaxel, the SP cells and the non-SP cells were further cultured for 24 hours with paclitaxel (5, 10 nM) and then, the cell viability was detected by MTT. To detect the effect of RA and FCRA^+^ on the sensitivity of SP cells to paclitaxel, the SP cells were cultured with RA and FCRA^+^ (5 μM) for 48 hours. Then, paclitaxel solutions (5 and 10 nM) were added and the cells were cultured for another 24 hours. Then, the cell viability was detected by MTT.

## 3. Results and discussion

The basic structure of ATRA consists of three parts: the hydrophobic part composed of three methylated ethylene units, the intermediate link of the conjugated four side chain, and the hydrophilic end made up of the carboxyl group. Carboxyl group is usually added to other groups to form retinoic acid derivatives, which also have anticancer activity.^27,28^ Ferrocene has excellent redox properties and can consume the anti-oxidant system in cancer cells; hence, it exhibits interesting cytotoxicity and anti-cancer activities.^29^ As shown in Fig. S1,† through the esterification reaction of retinoic acid and ferrocenylmethanol (FC), retinoic acid was added to a ferrocenyl group and hence, FCRA was synthesized. The ferrocene of FCRA can be oxidized to ferrocene cation (FCRA^+^) by FeCl_3_, which is sensitive to GSH and can be reduced to ferrocene by GSH. Considering that the increased content of intracellular GSH in cancer cells is related to the drug resistance, the consumption of GSH by FCRA^+^ may help reduce the drug resistance of cancer cells (Fig. S1†). Retinoic acid has a good effect on inhibiting proliferation and inducing differentiation of CSCs, but the ability of retinoic acid to induce apoptosis of cancer cells is poor. So the compound FCRA^+^ is expected that can enhance the ability of inducing apoptosis through depleting GSH in cancer cells, and the ability of inhibiting proliferation and inducing differentiation of CSCs will not be changed.

**Fig. 1:**
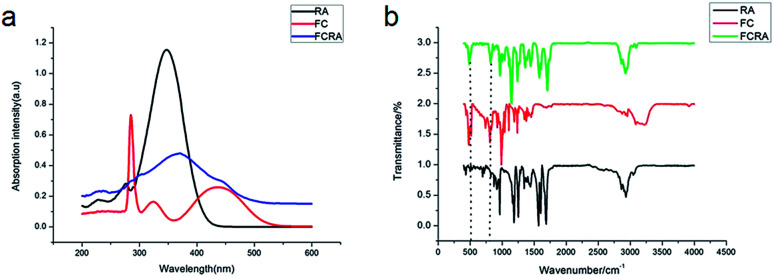
UV-vis spectra and FT-IR spectra of the FCRA.

We further tested the binding efficiency of FCRA by absorption and FTIR analysis. In the UV-vis spectrum of dichloromethane solution of FCRA, the wide peak was centered at *ca.* 370 nm (Fig. 1), which was different from that of RA and FC. These results indicated that FCRA is a new compound different from RA and FC. In the FTIR analysis of FCRA, the stretching vibrations of FCRA were observed at the same positions as that of RA from 900 cm^−1^ to 3250 cm^−1^, while at 500 cm^−1^ and 800 cm^−1^, the stretching vibrations of FCRA were the same as that of FC (Fig. 1). The FTIR results showed that FCRA had the chemical structures of FC and RA. In the ^1^H-NMR spectra, the absorption peaks at 4.0–4.5 ppm were ascribed to hydrogen on ferrocene. Moreover, the hydrogen peaks on COOH of RA near 12.5 ppm were absent, but the hydrogen peaks of other structures in RA were still retained (Fig. S2†). These results indicated that the product was dehydrated by the hydroxyl group of RA and carboxylic group of FC.

**Fig. 2:**
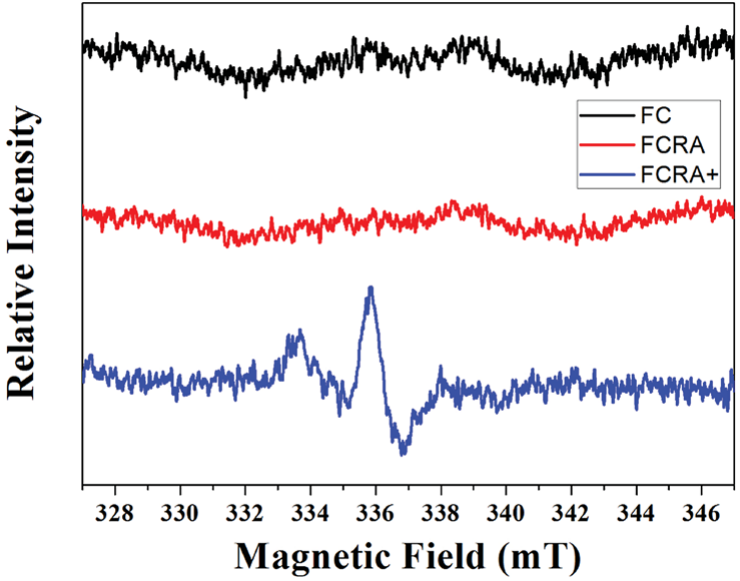
EPR spectra of FC, FCRA and FCRA^+^. FCRA^+^ had obvious stretching vibrations at 336 mT, and no obvious stretching vibrations were observed in FC and FCRA.

RA is a hydrophobic molecule , which limits its application, while the as-prepared FCRA has solved this problem. We additionally employed the electrochemical route to test the reduction ability of our as-prepared conjugate. The ferrocenyl group of FCRA can be oxidized by FeCl_3_ to ferrocenium, which is hydrophilic. Therefore, the water solubility of FCRA greatly increased, which could extend its application scope. As shown in the Fig. S3,† when the ratio (FCRA : FeCl_3_) reached 1 : 6, the redox potentials became stable, proving that all the ferrocenyl groups were oxidized to ferrocenium. When oxidized by excessive FeCl_3_, FCRA changed from insoluble suspension to transparent aqueous solution and the color also deepened. The distinct color changes of the drug conjugate depends on whether FC was successfully bound to RA. We further analyzed the elements and recorded the EPR spectrum of the FCRA^+^. Elemental analysis showed that the carbon content in FCRA^+^ was 42.11737% and the hydrogen content was 4.41217%. According to the content given by elemental analysis, the ratio of the number of atoms of carbon to hydrogen in the FCRA^+^ was calculated as 1 : 1.24831; in other words, C : H = 31 : 38.69761. In the proposed structure, the ratio was 31 : 38, which was very close to the result of element analysis. The results indicated that the FCRA^+^ accords with the proposed structural diagram (Fig. S1†). In the EPR spectra, no distinct stretching vibrations were observed in FC and FCRA, but distinct stretching vibrations at 336 mT were observed in FCRA^+^, as shown in Fig. 2. These results suggested that the ferrocenyl group of FCRA was oxidized by FeCl_3_ to ferrocenium, which had a free electron. The ferrocenium cation is sensitive to GSH;^30,31^ hence, it can deplete GSH in cancer cells to promote the apoptosis when FCRA^+^ enters the cancer cells.

**Fig. 3:**
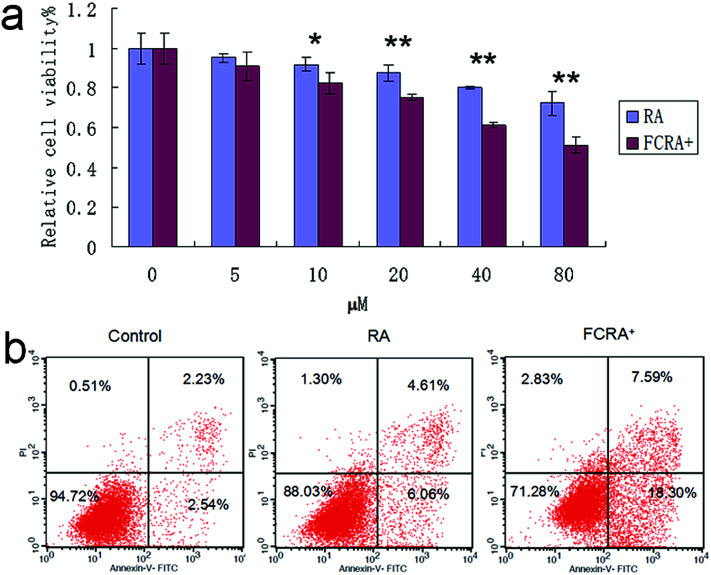
(a) Anti-cancer effect on melanoma cells of FCRA^+^ and RA by MTT assay; the IC50 of RA and FCRA^+^ were 123.7 μM and 53.43 μM respectively (*P < 0.05, **P < 0.01). (b) The cell death and apoptosis of A375 cells after staining with annexin V-FITC and propidium iodide (PI) by flow cytometry.

Human melanoma cells (A375) were used to evaluate the anticancer efficiency of RA and FCRA^+^ by methylthiazolyldiphenyl-tetrazolium bromide (MTT) assay. As shown in Fig. 3a, FCRA^+^ displayed stronger growth inhibitory effects to melanoma A375 cells, with the estimated IC50 values of 53.43 μM. RA was less effective against the growth of A375 cells, with the estimated IC50 values of 123.7 μM. Annexin V-FITC and PI double staining and flow cytometry were employed to evaluate the effect of RA and FCRA^+^ on inducing apoptosis. As shown in Fig. 3b, the cell death and apoptosis of cells incubated with RA was 11.97%, whereas that with FCRA^+^ reached 28.72%. These results indicated that FCRA^+^ was more capable of inducing melanoma cells apoptosis.

**Fig. 4:**
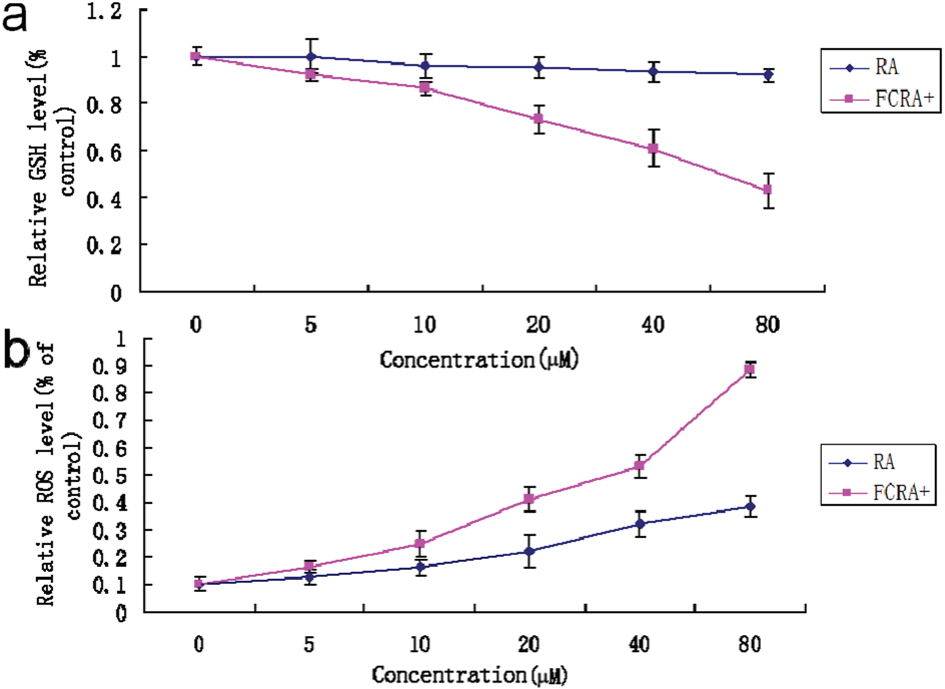
Effect of RA and FCRA^+^ on cellular GSH (a) of ROS (b) level of A375 cells. FCRA^+^ can deplete intracellular GSH, thus breaking down the redox homeostasis of melanoma cells, resulting in excessive accumulation of ROS. Thus FCRA^+^ shows better anti-cancer effect than RA.

Cancer cells exhibit persistently high reactive oxygen species (ROS) levels as a consequence of genetic, metabolic and microenvironment-associated alterations, which are then compensated by an increase in antioxidant ability from these cancer cells.^32^ Recent evidences indicate that alteration of this redox balance, which is a common hallmark of cancers, can be strongly implicated in malignant tumor progression and resistance to treatment.^33^ GSH is the major factor of intracellular antioxidant system in cancer cells. Thus, the depletion of GSH is considered an effective strategy for treating cancer. The cellular GSH and ROS level of A375 cells was examined by flow cytometric analysis after being treated with RA and FCRA^+^ for 24 h. We found that with the increase in drug concentration, the content of GSH in A375 cells treated by FCRA^+^ decreased significantly, and the accumulation of ROS significantly increased. In sharp contrast, free RA had no evident effect on the content of GSH in cells, and the level of ROS increased slightly (Fig. 4). These results indicated that the better anticancer effect of FCRA^+^ than RA was probably due to the disruption of redox homeostasis through depletion of GSH by FCRA^+^.

**Fig. 5:**
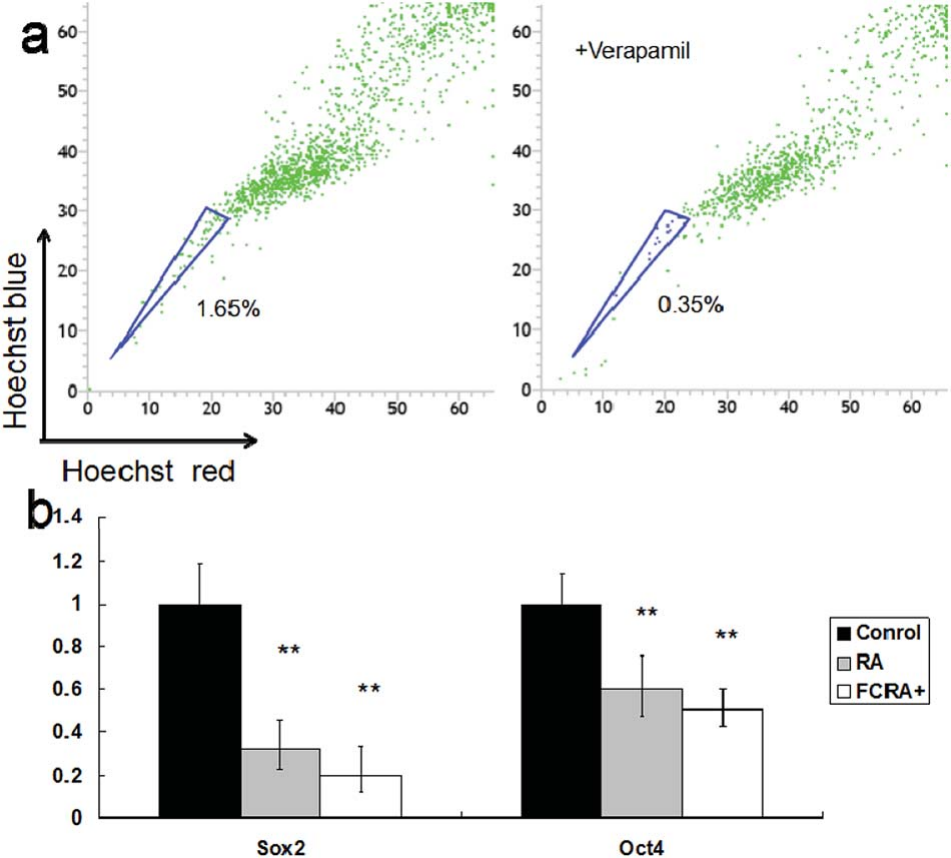
(a) Identification and sorting of the cancer stem cells (side population cells, SP cells) in human melanoma cell line A375. The cells were stained with Hoechst 33342 together with or without verapamil. (b) Expression of Oct4 and Sox2 in sorted SP cells before and after RA or FCRA^+^ treated for 6 h.

Although the ability of inducing apoptosis is weak, RA has a good ability to induce differentiation of CSCs into differentiated cancer cells (non-CSCs), which are more sensitive to chemotherapeutic drugs than CSCs; this is particularly critical for inhibiting drug resistance resulting from CSCs.^34^ Hoechst 33342 dual wavelength fluorescence analysis has been proven to be an effective tool in the purification and characterization of CSCs, and the Hoechst un-stained cells were described as the side population (SP) cells, namely, the CSCs.^35^ The melanoma CSCs were sorted from A375 cells by FACS Diva software. These results indicated that the percentage of CSCs (SP cells) was 1.65%. The ratio of CSCs decreased significantly on treating with verapamil, which has been reported as an inhibitor of Hoechst 33342 efflux^36^ (Fig. 5a). The expression of Oct4 and Sox2 in CSCs is closely related to their proliferation and differentiation.^37,38^ High expression of the genes promotes proliferation and inhibits differentiation of cancer stem cells. Conversely, low expression inhibits proliferation and promotes differentiation of CSCs. These results suggested that similar to RA, FCRA^+^ also inhibited the expression of Oct4 and Sox2 (Fig. 5b), which indicated that FCRA^+^ also has the same role as RA in inducing differentiation of A375 melanoma CSCs. We further detected the sensitivity of the melanoma CSCs to paclitaxel after RA and FCRA pretreatment. As shown in Fig. S4a,† CSCs of A375 cells (SP cells) were more tolerant to paclitaxel than non-CSCs (non-SP cell). When pretreated with RA and FCRA^+^ (5 μM for 48 h), the CSCs were more sensitive to paclitaxel than CSCs if not pretreated. Also, it seemed that FCRA^+^ was more capable of increasing the sensitivity of the CSCs to Paclitaxel than RA (Fig. S4b†). These results suggested that through pretreatment by RA and FCRA^+^, CSCs were differentiated and their sensitivity to paclitaxel increased. By binding to its nuclear receptor (RAR or RXR), RA regulates the transcription of target genes, such as Oct4 and Sox2, and inhibits the proliferation and promotes differentiation of stem cells.^39^ FCRA^+^ has the same functional structure of RA. Hence, it was supposed that the mechanism of FCRA^+^ regulating target gene may still be the binding of FCRA^+^ to RAR or RXR nuclear receptors. Moreover, FCRA^+^ has a stronger effect than RA on increasing the sensitivity of SP cells to paclitaxel, which may be related to the consumption of GSH in cells by FCRA^+^.

## 4. Conclusion

In summary, owing to poor water-solubility and ability to induce cancer cell apoptosis, the clinical use of retinoic acid is limited. Therefore, FCRA, through binding ferrocenylmethanol to *trans*-retinoic acid, was developed with highly improved water-solubility and anti-cancer ability. The ferrocenyl group of FCRA can be oxidized by ferric chloride (FeCl_3_) to ferrocenium, which is hydrophilic; hence, the water solubility of FCRA^+^ was greatly increased. FCRA^+^ has the same role as RA, that is, to induce the differentiation of melanoma stem cells. Also FCRA^+^ exerts stronger ability to inhibit the growth and induce apoptosis of melanoma cells by depleting GSH in the melanoma cells. Therefore, it may replace the clinical application of RA and has better application prospects.

## Conflicts of interest

There are no conflicts to declare.

## Supplementary Material

RA-008-C8RA04078H-s001
