# Supplementary material for: New ferrocene modified retinoic acid with enhanced efficacy against melanoma cells via GSH depletion
Source: RSC Adv. 2018 Aug 3;8(49):27740–5. doi: 10.1039/c8ra04078h (PMC9083447; doi:10.1039/c8ra04078h)
Supplement: RA-008-C8RA04078H-s001 [file RA-008-C8RA04078H-s001.pdf]

Electronic Supplementary Material (ESI) for RSC Advances.
This journal is © The Royal Society of Chemistry 2018

New ferrocene modified retinoic acid with enhanced efficacy against melanoma cells via GSH depletion

Yibo Wang[a,b], Bin Sun[b,c], Bin Han[b,c], Min Hu[a,b*]

[a] Department of orthodontics, School and Hospital of Stomatology, Jilin University, Changchun, 130041, P. R. China

[b] Jilin Provincial Key Laboratory of Tooth Development and Bone Remodeling, Jilin University, Changchun, 130041, P. R. China

[c] Department of oral and maxilloficial surgery, School and Hospital of Stomatology, Jilin University, Changchun, 130041, P. R. China

*Email: MinHu68@sohu.com

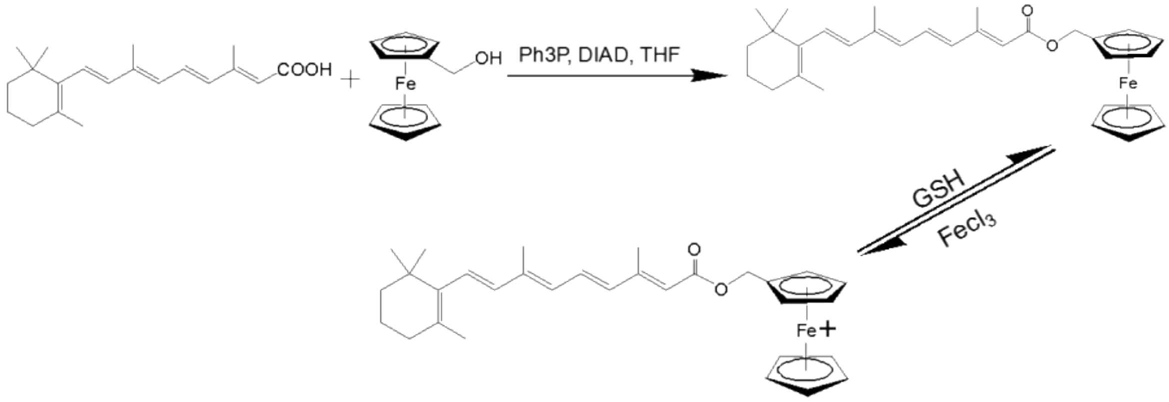

Figure S1. The chemical reaction equation of the synthesis of FCRA and FCRA^{+}.

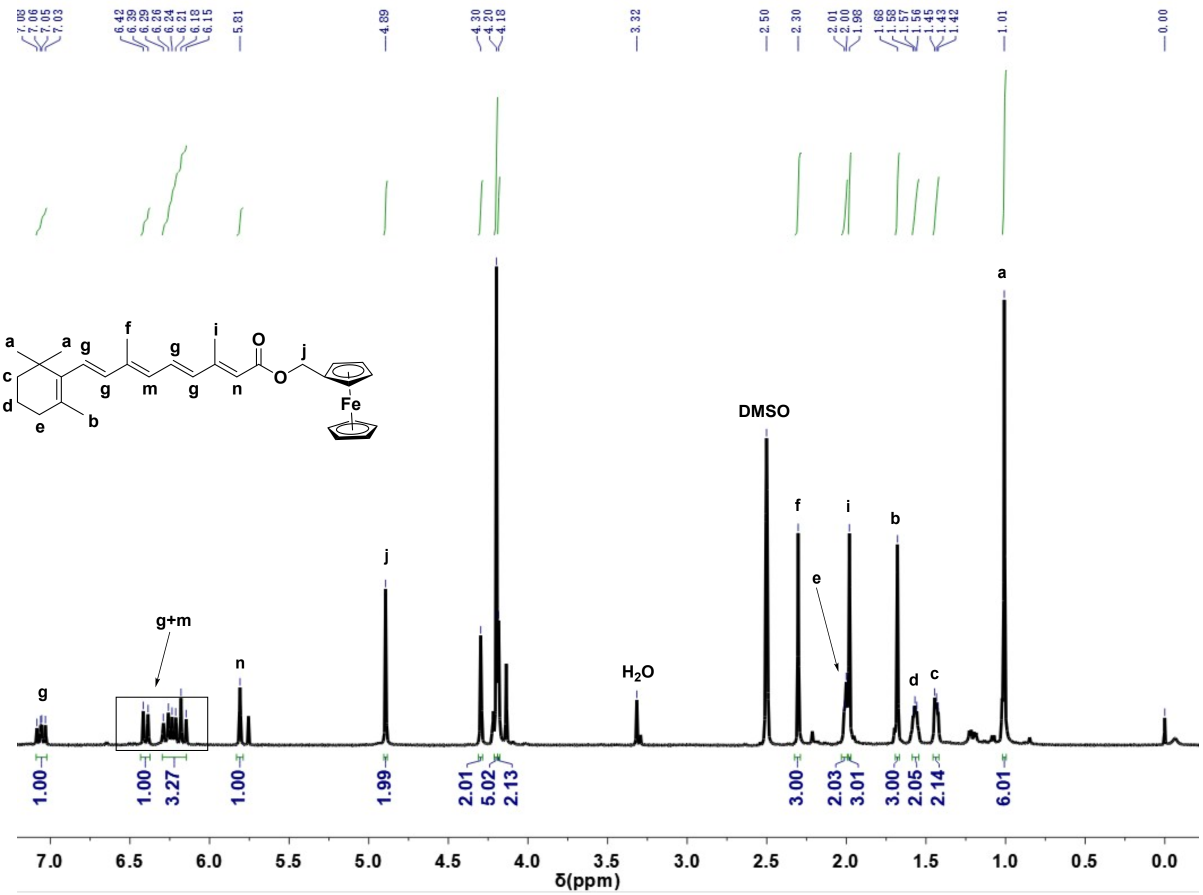

Figure S2. NMR spectra of FCRA. The absorption peaks at 4.0-4.5ppm were the absorption peak of hydrogen on the ferrocene, the hydrogen peaks on COOH of RA near 12.5ppm were disappeared, and the hydrogen peaks of other structures in RA were still retained.

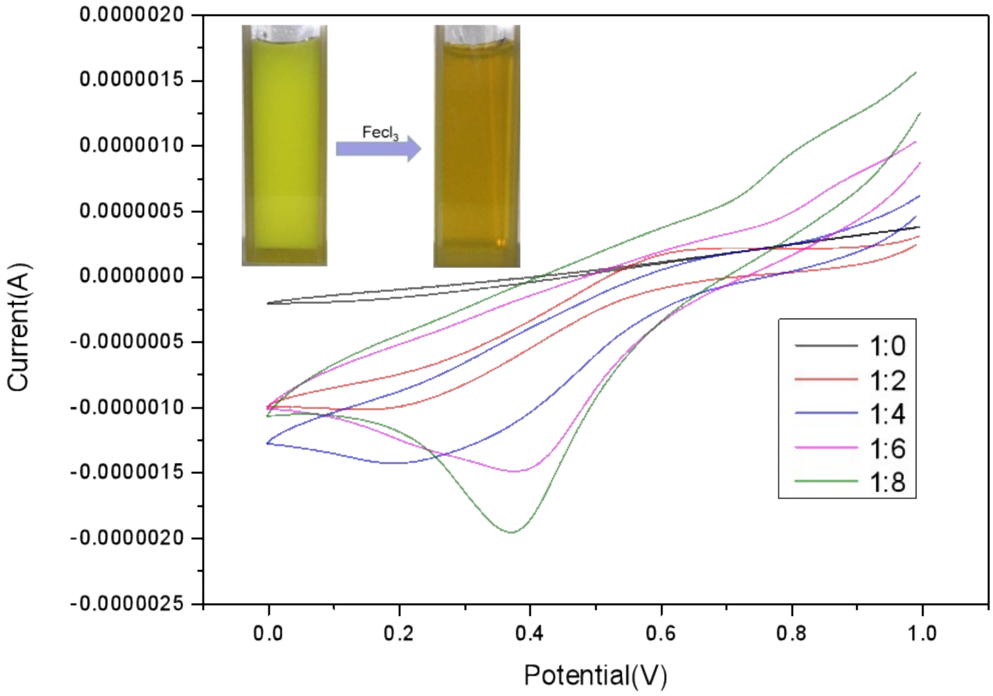

Figure S3. The electrochemical behavior of FCRA titrated by oxidant FeCl3 in CH2Cl2/CH3CN (v/v = 9:1). The inset images are the photograph of FCRA in the water solution before and after oxidizes by Fecl3.

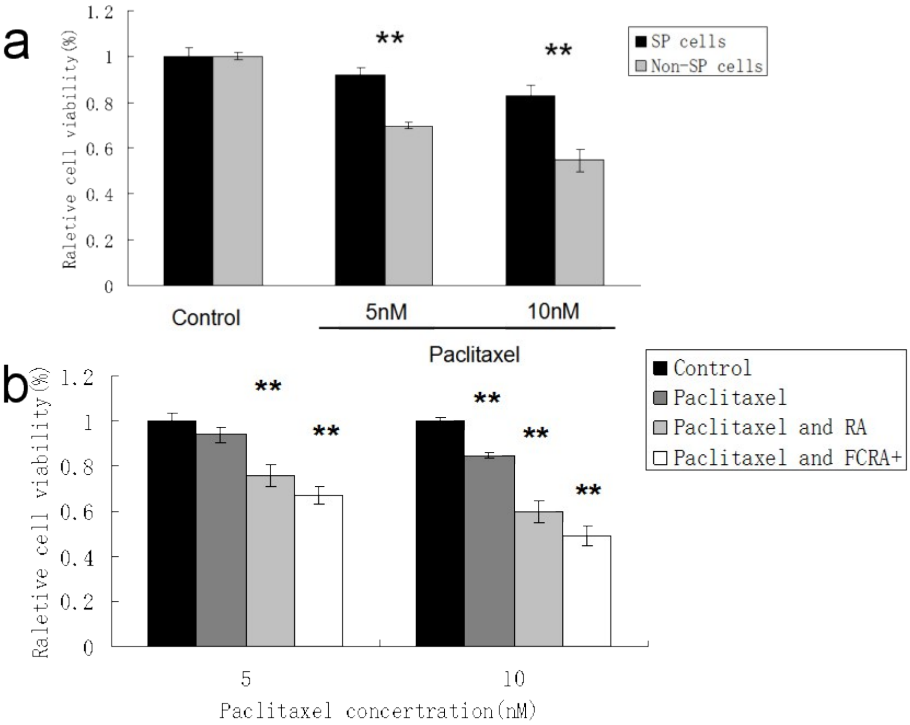

Figure S4. Sensitivity of the CSCs to Paclitaxel. a) SP cells of A375 cells (CSCs) were more tolerant to paclitaxel than non-SP cells (non CSCs)(** $P<0.01$). b) When pretreated with RA and FCRA$^+$(5 μ M for 48 h), the SP cells were more sensitive to paclitaxel than non pretreated SP cells(** $P<0.01$).
